# Association Mapping Reveals Novel Genetic Loci Contributing to Flooding Tolerance during Germination in *Indica* Rice

**DOI:** 10.3389/fpls.2017.00678

**Published:** 2017-04-25

**Authors:** Mengchen Zhang, Qing Lu, Wei Wu, Xiaojun Niu, Caihong Wang, Yue Feng, Qun Xu, Shan Wang, Xiaoping Yuan, Hanyong Yu, Yiping Wang, Xinghua Wei

**Affiliations:** ^1^State Key Laboratory of Rice Biology, China National Rice Research InstituteHangzhou, China; ^2^Crop Research Institute, Guangdong Academy of Agricultural Sciences (GAAS), South China Peanut Sub-Center of National Center of Oilseed Crops Improvement, Guangdong Key Laboratory for Crops Genetic ImprovementGuangzhou, China; ^3^Seeds Administration Center of Zhejiang ProvinceHangzhou, China

**Keywords:** genome-wide association study (GWAS), coleoptile, flooding, germination, rice (*Oryza sativa* L.)

## Abstract

Rice (*Oryza sativa* L.) is the only cereal crop that possesses the ability to germinate under flooded or other oxygen-deficient conditions. Rapid elongation of the coleoptile is a perfect response to flooding during germination, with coleoptile length differing among various rice varieties. Despite multiple studies have uncovered valuable information concerning this trait by focusing on the physiological metabolism of oxygen stress, the underlying genetic mechanism still remains unknown. In the present study, we screened coleoptile lengths of 432 *indica* varieties germinated in two environments (normal and flooded) and found more variation existing in flooded coleoptile length (FCL) rather than in normal coleoptile length (NCL). With the phenotypic data of NCL, FCL and FTI (flooding tolerance index), a genome-wide association study was performed by using 5291 single nucleotide polymorphism (SNP) markers. We detected 2, 11, and 9 significant SNPs under a mixed linear mode for NCL, FCL, and FTI, respectively. Of these SNPs, five were shared by FCL and FTI. Haplotype and phenotype effect analysis on the highest ranking locus indicated one of the two haplotypes contributed to coleoptile elongation remarkably. To better understand the controlling gene of this locus, reported expression profile data was applied. We focused on LOC_Os06g03520, a candidate gene which was highly induced by anoxia (∼507 fold). Sequence analysis in 51 varieties demonstrated Hap.2 associated perfectly with flooding tolerance. Further studies on this gene may help explore the molecular mechanism of rice flooding tolerance during germination. We believe our discoveries may conduce to isolating major genes and aid the improvement of flooding tolerance in modern breeding programs.

## Introduction

Flooding is a widespread, recurring problem that negatively affect rice (*Oryza sativa* L.) production in rain-fed lowlands areas of south and south-east Asia (Septiningsih et al., 2009). To respond to the oxygen-deficient stress caused by flooding, rice coleoptile develops fast to reach the surface of flooding water thus allow oxygen to transport to underwater tissues (Alpi and Beevers, 1983; Magneschi et al., 2009). This response is likely to be one of the most important patterns to study crop anaerobic metabolism.

Submergence caused by flooding creates a hypoxic environment that restricts plant aerobic respiration and leads to low ATP production. Originating in a semi-aquatic environment, rice has evolved a complicated response mechanism to adapt to long periods of submergence (Nagai et al., 2010; Kudahettige et al., 2011). The establishment of coleoptile at germination stage and highly developed aerenchyma in mature plants allow rice tissues to transport oxygen under water. Physiologically, various metabolic changes take place during flooding or oxygen-deficiency conditions. Enzymes related to starch degradation, glycolysis and ethanol fermentation, such as α-amylases, phosphofructokinase, fructose-6-phosphate-1-phosphotransferase, alcohol dehydrogenase and pyruvate dehydrogenase, are highly active under submerged conditions (Gibbs et al., 2000; Kato-Noguchi and Morokuma, 2007; Lasanthi-Kudahettige et al., 2007; Magneschi and Perata, 2009). Glucose also plays a positive role in flooding tolerance, with the exogenous application shown to restore this trait in intolerant type (Huang et al., 2003; Loreti et al., 2005). As a consequence, sugar and ATP concentrations can be maintained to provide basic energy for survival. In particularly, the high expression of *RAMY3D* and *ADH* correlated with the tolerance of flooding—an observation supported by numerous studies (Kato-Noguchi and Morokuma, 2007; Lasanthi-Kudahettige et al., 2007; Magneschi et al., 2009).

Genetic studies on rice germination under oxygen-deficiency condition mainly focus on exploring the controlling genes and their functional mechanisms. Numerous QTLs have been detected by using different genetic mapping populations. Angaji (2008) reported four putative QTLs on chromosomes 1, 2, 11, and 12 in a BC_2_F_2_ population derived from the cross of KHAIYAN and IR64. These QTLs explained 51.4% of the phenotypic variance. Similarly, Khao Hlan On, a tolerant accession, was crossed with IR64 to construct BC_2_F_2_ lines, and five QTLs were found on chromosomes 1, 3, 7, and 9 (Angaji et al., 2009). Septiningsih et al. (2013) unraveled six significant QTLs on chromosomes 2, 5, 6, and 7 by using a F_2:3_ population derived from IR42 and Ma-Zhan Red. These genetic loci would be valuable for further genetic mechanism researches.

Among the cloned genes related to flooding tolerance, *Sub1A*, one of the three ethylene-responsive factor (ERF) genes at *Sub1* locus, enables mature rice plants to survive 10–14 days under complete submergence stress (Fukao et al., 2006; Xu et al., 2006). With submergence, *Sub1A* significantly increases the GA signaling repressors Slender Rice-1 (SLR1) and SLR1 Like-1 (SLRL1) to suppress the GA-mediated growth regulation and diminishes other GA induced genes expression (Fukao and Bailey-Serres, 2008). Although *Sub1A* has major effect on submergence tolerance of mature rice plant, some rice cultivars lacking this locus, such as Nipponbare, display typical response to submergence during germination (Magneschi and Perata, 2009). This tolerance suggests that a *Sub1A*-independent mechanism may exist during rice germination under submergence (Lee et al., 2009). Ca^2+^ is a signal transducer during hypoxia and is also required for enzymes associated with glycolytic and ethanol fermentation in maize and Arabidopsis (Subbaiah et al., 1994; Sedbrook et al., 1996). The Calcineurin B-like (CBL) interacting protein kinases (CIPKs) has been found to act as key regulators of energy supply when oxygen availability is restricted, thus proving that Ca^2+^ signals play important roles in response to the oxygen stress (Batistic and Kudla, 2004). OsCIPK15, a CIPK family member, whose encoding gene is located on rice chromosome 12, functions in the sensing cascade associated with rice response to oxygen-deficiency (Lee et al., 2009). In rice, CIPK15 positively regulates sucrose non-fermenting 1 related protein kinase (SnRK1A) which is an essential regulator of MYBS1 (Halford et al., 2003; Lu et al., 2007). MYBS1 interacts with the sugar response complex (SRC) at the promoters of α-amylase encoding genes (Lu, 2002; Chen et al., 2006). The CIPK15-SnRK1A-MYBS1-α-amylase signaling pathway is suggested to be a critical response to sugar starvation when seeds suffer from submergence. *OsCIPK15* is also involved in the growth of the mature rice plants subjected to partial flooding (Lee et al., 2009; Kudahettige et al., 2011). *OsTPP7*, a gene encodes trehalose-6-phosphate phosphatase, was recently reported to enhance anaerobic germination tolerance in rice (Kretzschmar et al., 2015). Although the signal pathway of *OsTPP7* remains unclear at present, studies revealed that *OsTPP7* was involved in trehalose-6-phosphate metabolism and functions in enhancing starch mobilization to increase the tolerance of anaerobic germination.

With the first report of uncovering 14 agronomic traits in rice landraces, GWAS has become a popular strategy to dissect complex traits in rice (Huang et al., 2010). Compared with linkage mapping approach, association mapping possesses the ability to uncover a larger number of superior allele variations in broad natural populations, simultaneously in a simple and rapid pattern. Even though most published reports failed to identify new controlling gene, Yano et al. (2016) reported four novel genes influencing rice heading date, plant height, panicle number per plant and awn length with a single association study. These achievements rise new hope of uncovering novel genes with association mapping approach.

As the only organic response against oxygen-deficient stress, coleoptile elongation has become a reliable trait to study flooding tolerance of rice genotypes (Setter, 1994; Kato-Noguchi and Morokuma, 2007; Magneschi et al., 2009). In the present study, GWAS was performed on an *indica* population using reported SNP data (Lu et al., 2015), with respect to rice coleoptile elongation under normal and flooded environments. The objectives of this study were to: (a) detect the major genetic loci controlling rice flooding tolerance during germination; (b) explore favorable SNP alleles potentially useful for breeding; (c) find the most likely candidate gene. The results provide basic theory to enhance flooding tolerance in breeding program and also enable us to explore the genetic mechanism behind.

## Materials and Methods

### Plant Materials and Germination Experiment

On the basis of a previous study, we selected 432 *indica* accessions originating from 19 countries with 5291 SNPs genotyped by a custom-designed array (Lu et al., 2015) (Supplementary Table S1). Rice seeds were harvested in HZ (N 30°15′, E 120°12′), China, air-dried naturally, and maintained at 55°C for 5 days to break dormancy. Then seeds were surface-sterilized with 70% ethyl alcohol and washed three times with sterile water. For normal germination (control), plump seeds were wrapped in wet absorbent filter paper and vertically placed in a plastic box. For flooded germination, seeds were placed in a germination pouch and then submerged in sterile water to a 10-cm depth in a plastic box (O_2_ concentration was 0.12–0.13 mol m^-3^). Germination was allowed to proceed at 30°C in darkness for 10 days in an artificial climate chamber. Three replicated experiments were implemented for both control and flooded germination. After germination, coleoptile length was measured and data was accurate to millimeter.

### Statistical Analysis of Phenotypic Variation

The flooding tolerance index (FTI) was calculated as formula: flooded coleoptile length (FCL)/normal coleoptile length (NCL). Descriptive statistical analysis was performed in Microsoft Office Excel 2007. The analysis of variation (ANOVA) was carried out to evaluate the effects of genotype (G), and genotype × environment (G × E) using general linear model under SAS 9.0 (SAS, Inc., Cary, NC, USA). Variation and correlation analysis (SAS 9.0) were performed respectively to evaluate the coefficient of variation (CV) and estimate the relationship between NCL and FCL.

### Population Structure, Kinship Estimation

Principal component analysis was performed based on Nei’s genetic distance using Powermarker (ver. 3.25) (Liu and Muse, 2005) and NTSYSpc (ver. 2.1) (Rohlf, 2000). Pairwise relative kinship coefficients were calculated in TASSEL (ver. 4.0) (Bradbury et al., 2007).

### Association Mapping

Genome-wide association mapping was performed using TASSEL ver. 4.0 (Bradbury et al., 2007), and the EMMA (Kang et al., 2008) and P3D (Zhang et al., 2010) algorithms were used to reduce computing time. SNPs with a minimum count less than 75% of 432 individuals or with a minor allele frequency (MAF) less than 0.05 were removed from the association panel. Association analysis was implemented under a MLM which could be described as: *y* = *Xα*+ *Pβ* + *Kμ* + *e*, while *X* and *y* were vectors of genotype and phenotype data, *α* was the SNP effects, *β* represented the effects of population structure, *μ* was the vector of kinship background effects, e corresponded to residual effects, *P* was the PCA matrix relating *y* to *β, K* represented the related kinship matrix (Wen et al., 2014). The first three PCs were used to construct the *P* matrix in GWAS model.

### Gene Prediction and Verification

The LD heatmap around the significant SNPs in GWAS was constructed using the R package “LDheatmap” (Shin et al., 2006) and the candidate region was estimated using *r*^2^ > 0.6 (Yano et al., 2016). Candidate genes were predicted by screening the reference genome^1^. Online public expression profile data representing different induction of anoxic germination and aerobic germination were obtained from Rice eFP Brower of Bio-Analytic Resource for Plant Biology (BAR) database^2^. For qRT-PCR module, total RNA was extracted from rice coleoptiles after germination for 2 or 6 days using a MiniBEST Plant RNA Extraction kit (Takara Bio, Inc., Japan). First-strand cDNA was synthesized using PrimeScript RT Master Mix (Takara Bio Inc., Japan). To ensure cDNA quality following reverse transcription, a pair of primers straddling intron was used to detect genomic DNA. Then qPCR was performed in a two-step reaction using SYBR Premix Ex TaqII (Tli RNase Plus) (Takara Bio, Inc., Japan) on an Applied Biosystems 7500 Real-Time PCR system (Applied Biosystem, Carlsbad, CA, USA) with a pair of specific primers (CD3-f: atcaccttcaaccgccacat; CD3-r: gcggagtccatgtccatctg).

The full length of candidate gene and its promoter region were amplified by polymerase chain reaction (PCR) with two pairs of specific primers (QS14-f: tagatggatgagcacgcagc; QS14-r: gttccccctgcacaaacaac; QS15-f: gtaccacgaccaccatcttga; QS15-r: tgagctgatctcttggtcacg). Then PCR productions were purified and sequenced. All the sequences were edited and assembled using SeqMan in the package DNASTAR Lasergene (DNASTAR, Inc., Madison, WI, USA). Sequence analysis was performed using software MEGA6 (Tamura et al., 2013) and the haplotype analysis was carried out using DNAsp v5 (Librado and Rozas, 2009).

## Results

### Statistical Analysis of Phenotypic Variation

Totally, 432 *indica* varieties were screened for NCL and FCL (Supplementary Table S1). Coleoptile length ranged from 0.98 to 4.52 cm, with a mean value of 2.08 cm under normal germination condition (control). While the data ranged from 0 to 3.15 cm, with a mean value of 1.27 cm under flooded germination condition. The ANOVA indicated that both Genotype and Environment had significant effects on coleoptile lengths (**Table 1**). Moreover, the CV was 0.22 for NCL and 0.52 for FCL, revealing that more abundant variation existed in FCL. The correlation analysis indicated that there was merely low relationship between NCL and FCL (*R*^2^= 0.06, *P* = 0.2360). The FTI was calculated according to the ratio of phenotypic data of two environments. The results presented in **Table 1** showed that FTI ranged from 0 to 1.59, with a mean value of 0.65. The CV was 0.54, which was even higher than that of FCL.

**Table 1:** Statistical analysis of rice coleoptile length.

Environments	Mean (cm) ±*SE*	Range	CV	G × E
NCL	2.08 @ 0.001	0.98∼4.51 (cm)	0.2155	^∗∗∗^
FCL	1.27 @ 0.001	0.00∼3.15 (cm)	0.5248	
FTI	0.65 @ 0.0008	0∼1.60	0.5367	

*CV, coefficient of variation; NCL, normal coleoptile length; FCL, flooded coleoptile length; FTI, flooding tolerance index. ^∗∗∗^*P* < 0.001.*

### Population Structure and Relative Kinship

**FIGURE 1:**
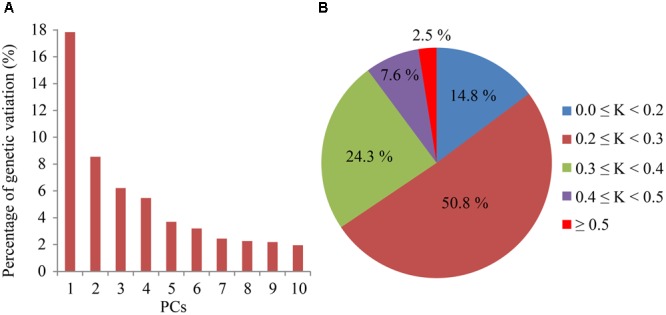
**Principal component analysis and relative kinship analysis of the *indica* population. (A)** The percentage of genetic variation explained by each of the first 10 principal components (PCs). **(B)** Distribution of relative kinship among 432 *indica* accessions, K represents relative kinship coefficients.

Considering that population structure may still exist in *indica* varieties and lead to false associations, we performed PCA based on *Nei’s* genetic distances (Supplementary Table S2). **Figure 1A** displayed genetic variation explained by the top 10 principle components (PCs) in bar chart. Most PCs explained very small proportion of genetic variation except the first one, suggesting a weak genetic structure of our population. According to our calculations, most varieties had no meaningful relationship except 2.5% of varieties possessed the kinship value larger than 0.5, which indicated the relative kinship of our population might have no strong influence on GWAS (**Figure 1B**). The analysis of genetic structure and relative kinship made it clear that our *indica* population was qualified for GWAS.

### Genome-Wide Association Mapping

**FIGURE 2:**
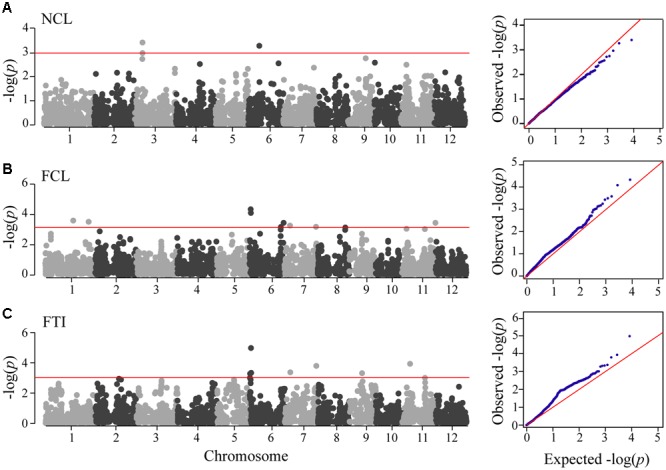
**Manhattan plots and quantile–quantile plots of genome-wide association analysis.** Manhattan plots, the red straight line show the threshold of *P* = 0.001; quantile–quantile plot, the red straight line represent the expected null distribution of *P*-values, the blue dots represent the observed distribution of *P*-values. **(A)** NCL, normal coleoptile length; **(B)** FCL, flooded coleoptile length; **(C)** FTI, flooding tolerance index.

Association mapping was performed under MLM with PC matrix and kinship matrix as covariates. At the thresholds of *P* = 0.001, we detected totally 22 significant SNP loci associated with NCL, FCL, and FTI (**Figure 2** and **Table 2**). Only two SNPs were detected under NCL, which were much less than the others. Moreover, none of these two SNPs were detected in the other two associations. These results confirmed that no correlation existed between the coleoptile length observed in control and flooded environments. The comparison of association between FCL and FTI identified five common SNPs, including two SNPs on chromosome 6, two SNPs on chromosome 7 and one SNP on chromosome 11 (**Figure 2**). These consistent loci revealed the similar contribution of FCL and FTI. The physical distance of two significant SNPs on chromosome 6 was only 211-kb, which suggested that they might belong to the same genetic loci. Considering its contribution to *P*-values and the steady identification by both FCL and FTI, this locus should be regarded as a major controller of rice flooded germination tolerance.

**Table 2:** Summary of significant SNPs detected by GWAS.

Traits	SNP markers	*P*-value	Chromosome	IRGSP.v4 position	IRGSP 1.0 position	Known loci
NCL	seq-rs1474	4.03E-04	3	7000314	6950989	
NCL	seq-rs2895	5.50E-04	6	10197319	10198318	
FCL	seq-rs408	2.66E-04	1	25073977	23417131	
FCL	seq-rs583	3.25E-04	1	39044291	37288444	Hsu and Tung, 2015
FCL	seq-rs2699	4.75E-05	6	1178093	1179092	
FCL	seq-rs2701	8.34E-05	6	1389722	1390721	
FCL	seq-rs3121	3.75E-04	6	30915718	30038716	
FCL	seq-rs3087	7.16E-04	6	28446724	27569722	
FCL	seq-rs3210	5.70E-04	7	5014710	4982558	Hsu and Tung, 2015
FCL	seq-rs3583	6.81E-04	7	28544962	27884804	qAG-7-2, Angaji et al., 2009; Hsu and Tung, 2015
FCL	seq-rs3972	7.58E-04	8	24927836	24839831	qAG-8-1, Angaji et al., 2009
FCL	seq-rs4773	9.33E-04	11	4581585	4598375	
FCL	seq-rs5125	9.81E-04	11	21084520	19256661	qAG-11, Jiang et al., 2006; Angaji et al., 2009
FTI	seq-rs2699	1.07E-05	6	1178093	1179092	
FTI	seq-rs2701	4.70E-04	6	1389722	1390721	
FTI	seq-rs2686	5.37E-04	6	534144	535143	
FTI	seq-rs2683	9.91E-04	6	276836	277836	
FTI	seq-rs3583	1.64E-04	7	28544962	27884804	qAG-7-2, Angaji et al., 2009; Hsu and Tung, 2015
FTI	seq-rs3210	4.30E-04	7	5014710	4982558	Hsu and Tung, 2015
FTI	seq-rs4216	4.88E-04	9	11439838	10874127	qAG-9-2, Kretzschmar et al., 2015
FTI	seq-rs4859	1.17E-04	11	7588747	7586818	
FTI	seq-rs5125	9.96E-04	11	21084520	19256719	qAG-11, Jiang et al., 2006; Angaji et al., 2009

To verify the accuracy of our results, we compared significant SNP loci in this study with reported QTLs which were detected previously using linkage or association mapping approach. As a consequence, some of our associated loci were not first detected in the research field of rice flooded germination tolerance (**Table 2**). The significant SNP seq-rs3210 on chromosome 7 in our study was found to locate closely to a genetic locus which was detected in another association study (Hsu and Tung, 2015). On chromosome 7, another significant SNP seq-rs3583 was identified in the genomic interval of qAG-7-2. This locus was also detected using both linkage mapping and association mapping approach (Angaji et al., 2009; Hsu and Tung, 2015), confirmed its real genetic effect. On chromosome 11, associated SNP seq-rs5120 was mapped in the genomic region of qAG-11 (Jiang et al., 2006; Angaji et al., 2009). In addition, SNP seq-rs4216, which was detected only in FTI, shared a very short distance with *OsTPP7* (Kretzschmar et al., 2015). Even though some of the co-localized genetic loci were previously detected using survival rates as the phenotypic indicator, they were also detected using FCL or FTI in this study.

### Analysis of Candidate Gene

**FIGURE 3:**
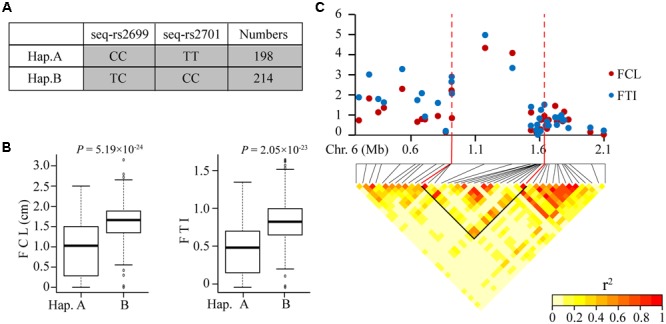
**Statistical analysis and candidate region estimation of seq-rs2699 and seq-rs2701. (A)** Haplotypes consist of the two significant SNPs, Numbers indicates the amounts of corresponding accessions; **(B)** Phenotypic effect of each haplotype; **(C)** Local manhattan plots and LD heatmap around the peak on chromosome 6, the candidate region estimated using *r*^2^ > 0.6.

The highest ranking of SNP, seq-rs2699 on the beginning position of chromosome 6, was firstly found to control rice FCL. In our study, this SNP was detected under both FCL and FTI. Detailed analysis on this locus was conducted to investigate the genetic mechanism behind. There were two significant SNPs on this locus which suggested the theoretic haplotypes should be at least four types. However, statistical analysis indicated that only two haplotypes accounted for most varieties (412 of the total 432 accessions) (**Figure 3A**). This result demonstrated the close linkage of these two significant SNPs. Subsequent calculation indicated phenotypic effect of these two haplotypes differed significantly. As was shown in **Figure 3B**, Hap.B associated with tolerant types significantly (with larger FCL and FTI). To accurately estimate the linkage region on the genomic map, pairwise LD correlations were calculated. With *r*^2^ = 0.6 as the threshold, we obtained a 875-kb candidate block containing seq-rs2699 and seq-rs2701 (**Figure 3C**). According to the reference genome, there were 158 genes in this region enabling us to isolate the most likely candidate gene.

To narrow down the candidate gene numbers, we took advantage of a previously reported expression profile which was obtained from a *japonica* cultivar with respect to normal and anoxic germination (Lasanthi-Kudahettige et al., 2007). With the use of these data we focused on a candidate gene, LOC_Os06g03520, which was annotated as DUF domain containing protein. The expression level of this gene was 507 times higher in coleoptile grown in anoxic environment than in which was grown in normal environment according to the expression profile. We then detected the relative expression level in flooded coleoptiles of four tolerant *indcia* varieties with normal coleoptiles as control (**Supplementary Figure S1**). The results observed in both four *indica* varieties, that LOC_Os06g03520 was highly expressed in flooded coleoptiles but not in normal coleoptiles after germination for 2 or 6 days, suggested its potential possibility for controlling rice FCL (**Supplementary Figure S1**).

**FIGURE 4:**
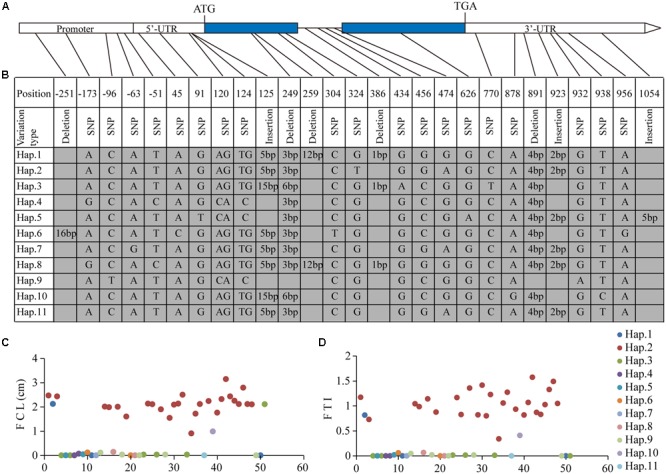
**Analysis of candidate gene LOC_Os06g03520. (A)** Gene structure of LOC_Os06g03520, the blue regions show the exons; **(B)** eleven haplotypes detected in 51 varieties with extreme phenotypes; **(C,D)** phenotypic distribution of the 51 varieties, x-axis indicates numbers sort by accessions ID; y-axis represents phenotypes. **(C)** FCL, flooded coleoptile length, **(D)** FTI, flooding tolerance index.

We then sequenced the full length and promoter region of LOC_Os06g03520 in varieties with extreme phenotypes (Supplementary Table S3). Totally, 27 polymorphism sites were identified including five sites located in exon regions (**Figure 4A**). Haplotype analysis revealed 11 haplotypes among these accessions (**Figure 4B**). To our surprise, almost every variety of Hap.2 was defined as tolerant type (with larger FCL and FTI) (**Figures 4C,D** and Supplementary Table S3), while the other haplotypes were mostly related to intolerant phenotypes. This result clearly demonstrated Hap.2 had a strong correlation relationship with flooding tolerance. Taken together, LOC_Os06g03520 was the most likely candidate gene controlling rice flooding tolerance during germination.

## Discussion

### Diverse Phenotypic Variation in *Indica* Population

One of the great necessities of studying flooding tolerance in *indica* subspecies is that most *indica* varieties are planted in low latitudes areas where they are more likely to suffer from flooding. The most sustainable methods to protect rice yields in face of such disasters is the use of flooding-tolerant cultivars (Angaji et al., 2009). We performed GWAS on an *indica* population and tried to detect the functional loci which could potentially use for breeding. Similar research has been reported before but with very small number of varieties and also lacking the evidence for targeted genes (Hsu and Tung, 2015). Our study with diverse *indica* varieties may enhance our knowledge for *indica* flooding tolerance. Previous researches reported that flooded coleoptiles of *japonica* varieties elongated faster than *indica* varieties (Lasanthi-Kudahettige et al., 2007; Hsu and Tung, 2015). However the difference of flooding tolerance inside *indica* subspecies still remains unknown. Our finding indicated varieties responded diversely to flooded germination in *indica* subspecies. This diversity would be beneficial to explore the genetic mechanism of flooding tolerance during *indica* rice germination.

Coleoptile elongation has been regarded as a major response to anaerobic stress (Setter, 1994; Lasanthi-Kudahettige et al., 2007; Magneschi et al., 2009; Kretzschmar et al., 2015). But few studies took variety characteristics into consideration. In this study, we found not all of the *indica* varieties respond to flooding by elongating coleoptiles rapidly. Some varieties displayed suppressed FCL compared with NCL. It can be inferred the molecular mechanism of these two responses were totally different. Our results of low correlation between NCL and FCL indicated flooding had triggered response and caused phenotypic changes. This demonstrated coleoptile elongation was strongly influenced by flooding. We tried to analyze the components inside coleoptile length. We assumed FCL was constituted of two components, the original part and the part induced by flooding. If we plan to study the response to flooding, the original part should be divided. However, the variation of coleoptile observed in normal environment was extremely low, which meant the part induced by flooding accounted for the majority of phenotypic variation. The common signals detected with FCL and FTI verified that they functioned similarly. Combine with the phenotypic variation analysis and the compute mode of FTI (FCL/NCL), we concluded that both FCL and FTI could equally represent rice response level to flooding.

### Known Genes Identified by GWAS

According to the results of GWAS, we have detected some SNPs close to known genes. For example, seq-rs3970 was localized close to the widely known oxygen-deficiency related gene – *OsRAMY3D*. The major function of *OsRAMY3D* is to promote starch amylolysis under energy deficiency (Setter, 1994; Gibbs et al., 2000; Magneschi and Perata, 2009; Nagai et al., 2010). The possible connection between *OsRAMY3D* and flooded germination tolerance can be inferred as well-according to our results. Kretzschmar et al. (2015) reported *OsTPP7* was involved in rice aneroarc tolerance. In tolerant type, *OsTPP7* accelerates coleoptile eglongation in oxygen-deficiency environment. We identified a sigificant SNP (seq-rs4216) close to *OsTPP7* at a distance of 1.4 MB. Because many SNPs around this gene were still not detected, we inferred that local LD might cause the misleading assocation and lead to the large distance.

### Idendified a Novel Candidate Gene Controlling Rice Flooding Tolerance

The differential expression profile strategy was an important approach to study genes associated with a given trait (Lamb et al., 2006). We took advantage of the reported expression profile data (Lasanthi-Kudahettige et al., 2007) and combined that with our association results. Within a novel detected locus, we identified a special gene might control flooded germination tolerance. Even though the expression profile data was collected using a *japonica* cultivar, the significant difference was also detected in four *indica* cultivars (**Supplementary Figure S1**). The subsequent analysis demonstrated our inference to be correct. The consistence of gene sequence and phenotype confrimed LOC_Os06g03520 was the most likely candidate. Unlike most genes controlling anerobic germnation was annoated to involve in energy metabolism pathway (*OsTPP7* and *OsCIPK15*), LOC_Os06g03520 was annoated as DUF domain containing protein. Untill now, more experiential evidence was required to verify its molecular function.

### Improve GWAS in Isolating Controlling Genes

Since its first report, GWAS has been widely carried out in rice genetic researches. Most studies were unable to identify candidate genes because of the large LD decay distance in rice. In our study, we used a gene chip carried 5291 SNP markers which was much less than those in other researches. Based on the consequence of Lu et al. (2015), most inter-marker distances were less than 100 kb, which was enough to cover the LD decay region. Recently, association mapping has been developed to isolated genes controling rice agronomic traits such as awn distribution, grain size, heading date, panicle number per plant (Si et al., 2016; Yano et al., 2016). The efficiency was proved to be amazingly high when Japanese scholars reported four novel genes using a signle GWAS (Yano et al., 2016). Because the denisty of our SNPs was not large enough to cover every candidate gene, we add sequence comparison of numerous individuals and expression profile analysis to confrim the result. We belive RNA-seq or transcriptome chip would be good techniques since they can guide researchers to a specific gene located in the association region.

## Author Contributions

MZ, QL, and XW: designed the research experiments. MZ, QL, and XN: performed the phenotyping. QX, YF, and CW: carried out the genetic studies. WW, XY, HY, and YW: managed the materials. XW: designed the overall project. MZ and QL: analyzed the data and drafted the manuscript. SW and CW: helped to revise the manuscript. All of the authors read and approved the final manuscript.

## Conflict of Interest Statement

The authors declare that the research was conducted in the absence of any commercial or financial relationships that could be construed as a potential conflict of interest.

## Footnotes

1 http://rice.plantbiology.msu.edu/

2 http://142.150.215.220/

## Supplementary Material

FIGURE S1 **Relative expression level of LOC_Os06g03520 detected in four *indcia* varieties and a *japonica* variety Nipponbare (NPB). (A)** Expression level in coleoptiles after germination for 2 days. **(B)** Expression level in coleoptiles after germination for 6 days.

## Abbreviations

FCL: flooded coleoptile length
FTI: flooding tolerance index
GWAS: genome-wide association study
LD: linkage disequilibrium
MLM: mixed linear model
NCL: normal coleoptile length
PCA: principal component analysis
QTL: quantitative trait locus
SNP: single nucleotide polymorphism.
