# Supplementary figures and images for: Association Mapping Reveals Novel Genetic Loci Contributing to Flooding Tolerance during Germination in Indica Rice
Source: Front Plant Sci. 2017 Apr 25;8:678. doi: 10.3389/fpls.2017.00678 (PMC5404183; doi:10.3389/fpls.2017.00678)

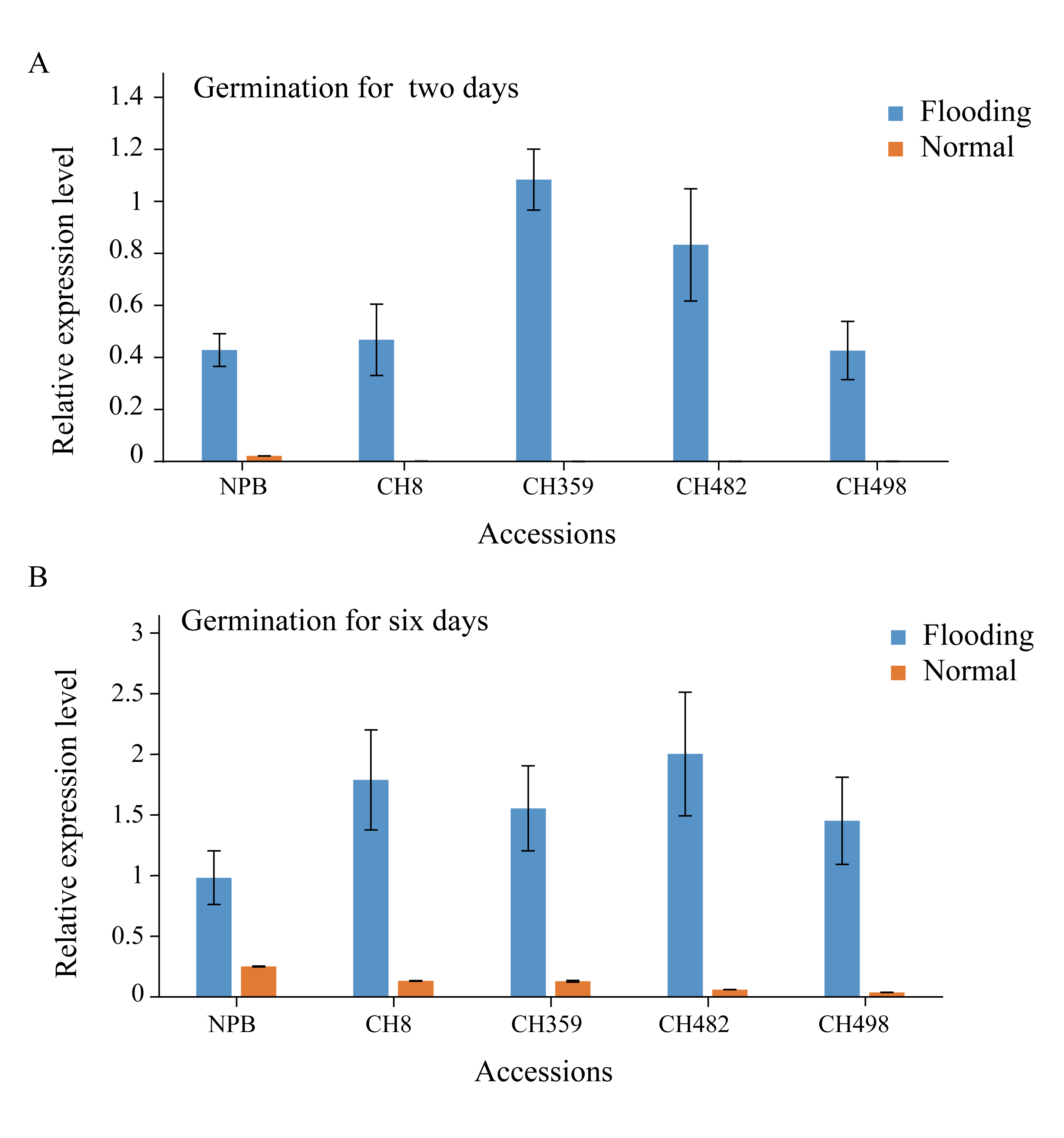

Supplement: FIGURE S1 — Relative expression level of LOC_Os06g03520 detected in four indcia varieties and a japonica variety Nipponbare (NPB). (A) Expression level in coleoptiles after germination for 2 days. (B) Expression level in coleoptiles after germination for 6 days. [file Image_1.TIF]
